# Children’s Self-Esteem and Attitudes toward Disability, Perceived Competence and Morality: The Indirect Effect of Cognitive Empathy

**DOI:** 10.3390/children9111705

**Published:** 2022-11-07

**Authors:** Alexandra Maftei

**Affiliations:** Faculty of Psychology and Education Sciences, Department of Education Sciences, Alexandru Ioan Cuza University, Iasi 700554, Romania; alexandra.maftei@uaic.ro

**Keywords:** disability, children, morality, competence, empathy, self-esteem

## Abstract

The present study explored children’s attitudes toward disability and the links with demographic factors (i.e., gender) and personal factors (i.e., empathy, sympathy, self-esteem). Our sample comprised 405 children aged 9 to 11 (M = 9.88, SD = 0.65, 47.4% males). First, we explored the links between self-esteem, empathy (cognitive and affective), and attitudes toward disability. Then, by using three scenarios involving a child in a wheelchair (Group 1), a child with an intellectual disability (Group 2), and a child with visual impairment (Group 3), we investigated the perceived competence and morality of these characters. The results suggested that cognitive empathy mediated the link between self-esteem and attitudes toward disability. Moreover, our data suggested that the character in a wheelchair (Group 1) received the highest scores regarding morality and competence, whereas the character with an intellectual disability (Group 2) received the lowest scores. We discuss the present findings regarding their practical implications for inclusive education strategies.

## 1. Introduction

A healthy society promotes positive attitudes toward individuals with disabilities and social inclusion for those individuals. Attitudes influence behavior, which influences knowledge, beliefs, and behavior. The most effective technique for changing children’s attitudes toward peers with disabilities is related to specific interventions aimed at increasing children’s knowledge about disabilities and providing exposure to peers with disabilities [1].

However, in a post-socialist Romania, still transitioning from decades of segregation against people with disabilities [2], children and adults with disabilities face discrimination and social exclusion, despite inclusive national policies adopted over the years, especially after joining the European Union [3]. During the past several years, Romania has acceded to most of the major international conventions that contain provisions regarding the rights of people with disabilities. International arrangements such as the UN Convention on the Rights of the Child (1990), the Jomtien Declaration on Education for All (1990), the Standard Rules for the Equalization of Opportunities for Persons with Disabilities (1993) and the Salamanca Convention (1994) were approved in a critical moment of change, to mark the beginning of an era focused on tolerance and respect for diversity [4,5]. 

Children’s attitudes toward disability are an essential factor to be explored to implement effective inclusive education programs and disability awareness campaigns aimed at reducing the discrimination and stigma toward people with disabilities [1], especially because children with disabilities are often subject to prejudice from their peers [6,7]. As previous studies suggested, it is crucial to understand these attitudes for timely, age-appropriate, and successful interventions to minimize stigmatization and social exclusion behaviors [8]. Thus, in the present study, we aimed to explore factors that might be linked to children’s attitudes toward disability, focusing on empathy and self-esteem. Moreover, given the findings that highlight the importance of the type of disability when discussing the perceived morality and competence of people with disabilities, we also investigated, by using three scenarios (each involving a different type of disability), the perceived competence and morality of these characters among the children in our sample.

## 2. Attitudes toward Disability: Gender, Age, and Previous Contact

Demographic factors are among the most investigated factors associated with people’s attitudes toward people with disabilities. However, previous studies have suggested ambivalent results regarding the gender and age of participants. For example, some studies suggested more positive attitudes from female participants, whereas others suggested no significant differences [9]. In addition, some studies suggested more positive attitudes for young participants toward people with disabilities [10,11], whereas others suggested the opposite [12], or no differences at all [13].

Babik and Gardner [6] explored the factors affecting the perception of disability from a developmental perspective. Their results suggested that, generally, the favorable attitudes toward disability of 3-year-old children become increasingly negative by 7–8 years, with negativity decreasing subsequently. Furthermore, the authors suggested that, with age, children rely more on experience than external instruction. Younger children may overgeneralize a situation and consider only the most salient aspects regarding a peer, for example, but older children can analyze a broader array of factors [14] and rely more on their experience. For instance, in a study by Kang and Inzlicht [15], 6–7-year-olds relied on external instruction when a negative, overt message contradicted their positive personal experience with an outgroup, whereas 10–11-year-olds trusted their own experience.

Furthermore, explicit teaching about prejudice, intergroup biases, and social justice reduces intergroup bias in 6–13-year-olds [16]. In addition, explicit intergroup prejudices seem to decrease with age due to social desirability concerns, as children become aware (by age 8) of social norms, usually reject prejudiced social judgments, and become driven to conform to those standards [17]. However, the implicit intergroup bias remains unaffected by social desirability pressures due to a lack of public responsibility [18].

Nevertheless, familiarity with disability and interaction with people with disabilities (e.g., friends or family members with a disability) are the strongest predictors of positive attitudes toward people with disabilities [19]. Direct exposure facilitates familiarity with people with disabilities and generally favors eliminating the related prejudices and stereotypes [20]. Furthermore, the lack of experience and contact with a particular condition might make it more difficult for people to offer assistance or create harmonious social ties with others impacted by the disability [19]. Preschool children, for example, are capable of harboring prejudices toward persons with distinguishing qualities from themselves. However, throughout this period, their attitudes are flexible and malleable, and they are quickly influenced by external variables such as the educational and sociocultural settings and family environment [21]. Therefore, the integration of people with disabilities into social groups is an essential component in the process of developing favorable attitudes among those who are close to these individuals. In addition, people can become more familiar with the particulars of a disability through frequent social interactions, which contributes to the development of their potential to assist people with disabilities [19]. At the same time, numerous social barriers brought about by a lack of knowledge and other misconceptions or prejudices can be addressed or enhanced through social contact between people with and without disabilities [22].

Finally, the research investigating children’s attitudes toward their peers with disabilities and the role of gender, age, and previous contact provided similar results as in adults. For example, Hong et al. [21] suggested that it may be beneficial for preschoolers to have more frequent interaction with persons with disabilities to develop favorable sentiments toward their peers with impairments, which is related to their understanding of disabilities. Similar findings were reported in the meta-analysis by Armstrong et al. [1], who suggested that programs involving direct contact, prolonged contact, and guided imagined interaction successfully improve children’s attitudes regarding disability.

## 3. The Type of Disability

Disability is often associated with erroneous preconceptions, stereotypes, and feelings of uncertainty. The construction of negative or maladaptive attitudes toward people with disabilities is often facilitated by a reduced familiarity related to the specifics of disabilities [22]. In addition, people generally have a limited understanding of the many different types of disabilities and, as a result, are frequently unclear about how they should behave when engaging with people with disabilities.

The type of disability has been previously explored in studies examining the different views regarding disability. For instance, Paseka and Schwab [23] suggested a higher level of favorable attitudes regarding the school integration of students diagnosed with physical disabilities. On the other hand, they observed less favorable attitudes toward integrating students diagnosed with behavioral disorders or intellectual deficiencies. In the research that Jury et al. [24] conducted among teachers, they found comparable results. More specifically, the authors suggested more positive attitudes toward children with motor disabilities and a significantly lower positive attitude toward students with cognitive impairments or autistic spectrum disorders.

Children’s attitudes and perceptions towards disabled persons are also subject to the type of disability. For example, some studies suggested that the more visible the impairment, the less favorable the attitudes [25]. However, other studies suggested the opposite, with children having more negative attitudes toward invisible disabilities (e.g., intellectual disability) than visible ones (e.g., a physical/motor disability) [26]. Finally, in their review, Babik and Gardner [6] suggested that children with emotional or behavioral problems, as well as those with multiple disabilities, are perceived unfavorably by their typical peers more than children with a specific physical disability. Furthermore, children with intellectual or physical/intellectual disabilities seem to be perceived more negatively than children with physical disabilities [27] the level of social inclusion being linked to the child’s mental age [28]. In other words, intellectual disability may be more salient to typical children than physical disability, particularly within the school context [6].

## 4. Empathy, Self-Esteem, and the Attitudes toward Disability

Empathy comprises the ability to feel and understand another person’s emotional state or condition through emotional matching and effect sharing and is essential for prosocial behavior, social competence, and moral thinking [29,30]. Cognitive empathy is the ability to perceive and understand another’s mental state (part of the theory of mind), whereas affective empathy is the ability to share others’ experiences without direct emotional stimulation [31]. In addition, affective empathy develops significantly earlier than cognitive empathy [32].

Empathic children understand better others’ feelings and are generally more likely to respond appropriately and sensitively, and help those in need, leading to better interpersonal relationships, prosocial behaviors (e.g., caring for others, relieving suffering, treating others with kindness), and moral reasoning in children and early adolescents [29,30,33,34]. In addition, positive interactions with peers with disabilities enhance typical children’s awareness of others’ emotions and might generate more accepting attitudes toward peers with disabilities [35]. Thus, in addition to contact and familiarity, empathy may improve children’s views toward outgroups, including peers with disabilities [6].

Self-esteem describes how much someone likes and values themselves [36] and might be one of the factors influencing the attitudes toward outgroups, including people with disabilities. The way one evaluates the ones around them can be viewed through the lens of self-esteem because others’ evaluations begin with self-evaluation [6]. Higher self-esteem is linked to better mental health and healthier relationships [37]. Although we know from previous studies that self-esteem is significantly associated with empathy [38] and that empathy is a significant factor related to children’s attitudes toward disability [6], the literature regarding the specific link between self-esteem and children’s attitudes toward disability is scarce; thus, one of the present research aims is to address this gap.

## 5. Perceived Morality and Competence of People with Disabilities

Most group stereotypes fall along dichotomous dimensions, namely agency and communality, competence and morality, or competence and warmth [39]. These stereotypes are based on social hierarchies, with high-status groups perceived as competent but cold and low-status groups as incompetent but warm. For example, the stereotypes about a group’s ability, intelligence, efficiency, and strength fall under competence. The warmth dimension includes social and moral qualities, such as trustworthiness, sincerity, communality, friendliness, and openness [40].

There are various combinations of warmth and competence perceptions that shape the attitudes toward people with disability. For example, a high warmth–low competence stereotype induces pity and causes either avoidance or overt support toward the individual with disabilities. Conversely, a high warmth–high competence stereotype generates appreciation and positive behavioral reactions, including a willingness to help and work with the individual with a disability [39]. However, most studies suggested that people with disabilities are often subject to competence-related stereotyping [39]. Generally, individuals with disabilities are perceived as less competent than their typical peers [40], leading to discriminatory behaviors and social inequity, because incompetence is a valid basis for discrimination [41]. Nevertheless, these findings resulted from research among adults, and little is known about children’s perceptions of the morality and competence of people with disabilities. Thus, our study aimed to address this gap.

## 6. The Present Study

Using a cross-sectional approach, the present study aimed to investigate children’s attitudes toward disability and the links with demographic factors (gender), contact (i.e., friends or family members with disabilities), and personal characteristics (i.e., empathy, sympathy, self-esteem). Also, we explored the potential indirect of empathy on the relationship between self-esteem and attitudes toward disability. Finally, we aimed to investigate and compare the perceived competence and morality of different characters with disabilities, depicted in experimental scenarios, following Smith and Williams [42].

Based on previous findings, our assumptions were the following. Hypothesis 1 states that female participants would express more positive attitudes toward disability than male participants. Hypothesis 2 states that empathy would mediate the link between self-esteem and children’s attitudes toward disability. Hypothesis 3 states that the type of disability would generate significant variability in children’s perception of morality and competence.

## 7. Method

### 7.1. Participants and Procedure

Our sample consisted of 405 typical children (i.e., with no disabilities) aged 9 (*N* = 114, 28.1%), 10 (*N* = 225, 55.6%), and 11 (*N* = 66, 16.3%), of which 192 (47.4%) were males and 213 (52.6%) were females. All children were students from different schools located on the northeastern side of Romania. The research protocol was designed following the ethical requirements specific to the faculty where the authors are affiliated, in accord with the 2013 Declaration of Helsinki (ethical approval number: 408/2022/ethical approval date: 14 February 2022). Before beginning the study, we distributed letters describing the study to the parents and school principals during parent–teacher meetings. The letters contained a short description of the study’s aim (i.e., to explore a series of personal factors related to the attitudes toward inclusive educational practices) and details related to the research procedure: (1) the confidentiality and anonymity of the answers, (2) the fact that all participating was voluntary and children can retire from the study at any point, and (3) the time needed to answer the scales’ items, i.e., around 20 min. Following parental approval (around 60% agreement rate), children answered the self-reported items in their regular classrooms on a regular school day.

### 7.2. Measures

First, we randomly distributed children into three groups that read three different scenarios adapted from the research conducted by Smith and Williams [42]. The scenarios described either a child in a wheelchair (Group 1), a child with an intellectual disability (Group 2), or a child with visual impairment (Group 3).

Attitudes toward disability. We used the 36-item Chedoke–McMaster Attitudes Towards Children with Handicaps Scale (CATCH scale; [43]) to measure children’s attitudes toward disability. We replaced the word “handicap” with “disability”, and example items included “I wouldn’t mind if a child with a disability sits next to me”, “Children with disabilities can do lots of things for themselves”, and “Children with disabilities like to play”. The self-reported items are measured on a Likert scale ranging from 0 (strongly disagree) to 4 (strongly agree). We chose this scale as it is considered one of the comprehensive measurements of attitudes toward disability [44], as it comprises emotions, behaviors, and cognitions related to disability, and it was previously used in a high number of related studies [27]. Moreover, the scale demonstrated acceptable internal consistency (a = 0.90) and test–retest stability [44]. Some studies used the three dimensions of the scale separately [45], whereas others did not support the multidimensionality of the instrument but rather its unidimensionality [46,47]. In the present study, we used the overall score of the scale, with higher scores indicating more favorable attitudes toward disability. Cronbach’s alpha in the present study was 0.70.

Empathy. We used the Adolescent Measure of Empathy and Sympathy-AMES [48] to measure children’s cognitive (4 items; 0.25 interitem correlation mean) and affective empathy (4 items; 0.33 interitem correlation mean). Example items included “I can easily tell how others are feeling” for cognitive empathy, and “When a friend is angry, I feel angry too” for affective empathy. Children answered the items on a 5-point Likert scale ranging from 1 (never) to 5 (always). Higher scores indicated higher empathy.

Self-esteem. We used the 10-item self-esteem scale developed by [49] to measure children’s self-reported self-esteem. Example items included “On the whole, I am satisfied with myself” and “I feel that I have a number of good qualities.” Children answered the items on a Likert scale ranging from 1 (strongly disagree) to 5 (strongly agree). Higher scores indicated higher self-esteem. Cronbach’s alpha in the present study was 0.66 (inter-item correlation mean = 0.16).

Finally, the children were asked to answer the following questions regarding the character’s morality and competence. The first question was (1) Morality: Do you think… (i.e., the character depicted in the scenario presented) is a moral person? (Yes/No/I don’t know). The second question was (2) Competence: Do you think… (i.e., the character depicted in the scenario presented) is a competent person? (Yes/No/I don’t know). To ensure all children understood morality and competency, the experimenters first explained the two concepts, and the children were also asked to offer examples of behaviors that would suggest either a moral or a competent person.

Finally, a demographic scale assessed participants’ gender, age, and whether they had a friend (Yes/No) or a family member with a disability (Yes/No/I don’t know). All the instruments were pretested in a sample of 16 children aged 9 to 10, and no issues were reported related to item understanding or any other potential issues. We employed the back-translation procedure to check the consistency of the quality of the translated research instruments, and we found no discrepancies [50]. A demographic scale assessed participants’ gender and age.

## 8. Results

### 8.1. Overview of the Statistical Analyses

We used the 24.0 version of the IBM SPSS and the SPSS macro program PROCESS [51] to analyze the data. We first calculated the descriptive statistics for all the main variables and subsequently conducted (1) T-test results to assess the potential gender difference and (2) bivariate correlation analyses to explore the relationships between the primary variables. The data cleaning steps included careless responding, failed attention checks, and outliers.

Table 1 describes the sample characteristics depending on the group they were randomly assigned to.

We further proceeded with parametric tests, given the results (see Table 2) suggesting the normality of our distribution (skewness and kurtosis values between −1 and =1 [52]. Next, we performed independent T-tests to investigate the potential gender differences regarding the main variables. We found significant differences, *t* (403), *p* = 0.02, regarding affective empathy, with girls (M = 10.34) scoring significantly higher than boys (M = 9.56). However, no significant differences emerged regarding the overall attitude toward disability, cognitive empathy, and self-esteem (all *p*-s > 0.05).

We further computed zero-order correlations, and the results are presented in Table 3. Our data suggested positive associations between the attitudes toward disability and cognitive empathy (*r* = 0.19, *p* < 0.001) and age (*r* = 0.09, *p* = 0.04). In addition, cognitive empathy was positively related to affective empathy (*r* = 0.11, *p* = 0.01), self-esteem (*r* = 0.14, *p* = 0.003), and age (*r* = 0.13, *p* = 0.007). Finally, we found a negative association between affective empathy and self-esteem (*r* = −0.24, *p* < 0.001).

Next, based on the correlation analysis results, we performed a linear regression analysis to explore how much variance of the attitudes toward disability was explained by participants’ age and cognitive empathy. Results suggested that the proposed predictive model was significant, *F* (2, 402) = 8.94, *p* < 0.001. Together, age (β = 0.07, *p* = 0.13) and cognitive empathy (β = 0.18, *p* < 0.001) explained 4.3% of the variance in children’s attitudes toward disability (though cognitive empathy was the only significant predictor).

### 8.2. The Indirect Effect of Self-Esteem through Cognitive Empathy

**Figure 1:**
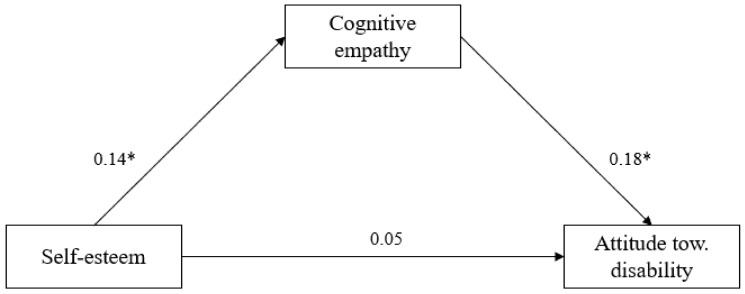
The indirect effect of cognitive empathy on the link between self-esteem and children’s attitudes toward disability (*N* = 405). The values represent standardized coefficients. * *p* < 0.05.

Finally, we tested whether self-esteem predicted attitudes toward disability through cognitive empathy. The mediation analyses were performed by using the SPSS macro program PROCESS (Model 4, 95% confidence interval (CI) with 5000 bootstrapped samples). The total effect of self-esteem (i.e., without considering the mediator) was not significant; *b* = 0.22, *SE* = 0.13, 95% CI [−0.04; 0.49], *R*^2^ = 0.006. The effect of self-esteem on cognitive was significant; *b* = 0.08, *SE* = 0.02, 95% CI [.02; 0.14], *R*^2^ = 0.02. In the model that included both self-esteem and cognitive empathy, self-esteem did not emerge as a significant predictor of the attitudes toward disability, *b* = 0.14, *SE* = 139, 95% CI [−0.12; 0.41]. The direct effect of self-esteem on the attitudes toward disability was not significant in this model, *b* = 0.14, *SE* = 0.13, 95% CI [−0.12; 0.41], but the indirect effect was: *b* = 0.07, *SE* = 0.03, 95% CI [0.02; 0.15]. Therefore, cognitive empathy fully mediated the link between self-esteem and children’s attitudes toward disability (see Figure 1). The same significance of the relationships between the variables was maintained when controlling for gender and age.

### 8.3. Perceived Competence and Morality

Next, we explored the perceived competence and morality of the characters with a disability in all three groups (see Table 4). We coded children’s answers with 0 (No/I don’t know) and 1 (Yes). Results suggested that the character presented in the vignette from Group 1 (i.e., the child in a wheelchair) received the highest score regarding perceived competence and morality. Conversely, the lowest score regarding competence and morality was reported in Group 2 (intellectual disability).

## 9. Discussion

People with disabilities frequently experience barriers when seeking public services, education, or employment. In addition, the disparity in services access between those with and without disabilities can often lead to hurdles to social integration for those with various disability diagnoses. Public attitudes toward people with impairments can either maintain these barriers or, on the other hand, ameliorate and ultimately erase them. Therefore, as the literature demonstrates, positive attitudes toward disability can help to facilitate and more effectively integrate people with disabilities and combat their discrimination and stereotyping, whereas negative attitudes can either directly or indirectly encourage hostile behaviors through neglect or the formation of false beliefs about the morality or competence of people with disabilities. In this context, the present study adds to the literature regarding children’s attitudes toward disability, emphasizing the importance of self-esteem and empathy.

Our results suggested that cognitive empathy (and not affective empathy) fully mediated the link between self-esteem and children’s attitudes toward disability, regardless of gender and age. In other words, we found that children’s self-esteem- which, according to previous literature, might work as a mirror for how we treat others [6], seems to predict children’s cognitive empathy, which predicts their attitudes toward disability. Cognitive empathy describes the ability to recognize and understand another’s mental state—in our case, peers with disabilities.

Regarding the perceived competence and morality of the characters with disabilities depicted in the scenarios we used, our data suggested that the character in a wheelchair received the highest score regarding perceived competence and morality. In contrast, the lowest score regarding competence and morality was reported concerning the character with an intellectual disability. Our data aligns with previous studies suggesting that people with physical disabilities might generally be perceived more positively than those with intellectual disabilities [23,24]. At the same time, our data contradict previous studies suggesting that the more visible the impairment (i.e., wheelchair), the less favorable the attitudes [25]. On the other hand, our data support other studies suggesting that children hold more negative attitudes toward invisible disabilities, such as intellectual disabilities, compared to more visible ones, such as physical/motor disabilities [6,26,27].

From a more practical point of view, our results call for inclusive approaches aimed at increasing the knowledge and contact about disability, which might also lead to higher cognitive empathy and, thus, more positive attitudes toward disability. Our data highlights the importance of children’s self-esteem in processing and understanding others’ emotions. More specifically, our data indicated that children’s self-esteem predicts how they can understand and perceive others’ emotions, and this significant indirect effect regarding disability is significant through its practical implications. For example, the present results highlight the need to promote and enhance children’s self-esteem to build inclusive, healthy societies because this seems to lead to more empathic citizens and more positive attitudes toward disability. In addition, our results point to the fact that understanding others’ emotions, and not just mirroring what a person feels, is also important when shaping inclusive education practices (since cognitive and not affective empathy mediated the link between self-esteem and attitudes toward disability).

Some limitations need to be mentioned regarding the present research. First, the sample of participants was relatively small, limiting the generalizability of our findings. Secondly, the cross-sectional nature of our study impedes us from drawing any conclusions related to the causal relationship between the variables. Future studies would benefit from longitudinal measurements, especially when discussing the indirect effect of cognitive empathy. Thirdly, regarding the experimental procedure, we did not use a control group, limiting our findings’ nature.

Furthermore, we only used intergroup comparisons and not in-group designs; thus, we could not compare the perceived competence and morality regarding the proposed characters. Future studies might benefit from extending these results by using in-group methodological approaches. Finally, several other variables might have determined significant variances in children’s answers, such as parental and attachment styles and various societal factors [6]. Moreover, exploring the family-related characteristics (for example, parental education, economic status, the presence of siblings with disabilities), as well as prosocial behavioral patterns might reveal other significant associations related to participants’ self-esteem, cognitive and affective empathy, and attitudes toward disability. Though the present study did not aim to investigate these influences, it is essential to acknowledge their importance and interpret the current findings accordingly.

## 10. Conclusions

Despite these limitations, our results shed more light on the role of self-esteem and cognitive empathy in shaping children’s attitudes toward disability, highlighting the need to enhance children’s self-esteem and promote empathy to shape more positive attitudes toward disability. Finally, perhaps the most important outcome of the present research is the finding that positive attitudes toward others start with positive attitudes toward ourselves.

## Figures and Tables

**Table 1:** Sample characteristics (depending on the experimental group).

Group	Age (*M/SD*)	Gender: Male (*N*/%)	Gender: Female (*N*/%)	Friend w/ Disabilities: Yes (*N*/%)	Friend w/ Disabilities: *N*o (*N*/%)	Friend w/ Disabilities: I Don’t Know (*N*/%)	Family Member w/ Disabilities: Yes (*N*/%)	Family Member w/ Disabilities: *N*o (*N*/%)	Family Member w/ Disabilities: I Don’t Know (*N*/%)
1 (*N* = 136)	9.88 (0.60)	*N* = 62 (45.6)	*N* = 74 (54.4)	*N* = 62 (45.6)	*N* = 121 (89)	0	*N* = 10 (7.4)	*N* = 115 (84.6)	*N* = 11 (8.1)
2 (*N* = 136)	9.92 (0.65)	*N* = 62 (45.6)	*N* = 74 (54.4)	*N* = 6 (4.4)	*N* = 123 (90.4)	*N* = 7 (5.1)	*N* = 18 (13.2)	*N* = 106 (77.9)	*N* = 12 (8.8)
3 (*N* = 133)	9.82 (0.71)	*N* = 77 (57.9)	*N* = 56 (42.1)	*N* = 4 (3.0)	*N* = 118 (88.7)	*N* = 10 (7.5)	*N* = 5 (3.8)	*N* = 118 (88.7)	*N* = 10 (7.5)

**Table 2:** Descriptive statistics for the main variables (*N* = 405).

Variable	M	SD	Min	Max	Skewness	Kurtosis
CATCH (overall)	50.03	12.19	9	103	−0.07	0.23
Cognitive empathy	15.22	2.57	7	20	−0.36	−0.24
Affective empathy	9.97	3.44	4	20	0.23	−0.43
Self-esteem	29.37	4.40	13	40	−0.28	0.40

**Table 3:** Zero-order correlations between the main variables (*N* = 405).

Variable	1	2	3	4
1. CATCH (overall)	-			
2. Cognitive empathy	0.19 **	-		
3. Affective empathy	0.07	0.11 *	-	
4. Self-esteem	0.08	0.14 *	−0.24 **	-
5. Age	0.09 *	0.13 *	0.09	−0.07

** *p* < 0.001; * *p* < 0.05.

**Table 4:** Perceived competence and morality (*N* = 405).

Group	Perceived Competence			Perceived Morality		
	Yes (n/%)	No/I Don’t Know (n/%)	M (SD)	Yes (n/%)	No/I Don’t Know (n/%)	M (SD)
1 (*N* = 136)	128 (94.1)	8 (5.9)	0.94 (0.23)	130 (95.6)	6 (4.4)	0.95 (0.20)
2 (*N* = 136)	85 (62.5)	51 (37.5)	0.62 (0.48)	126 (92.6)	10 (7.4)	0.92 (0.26)
3 (*N* = 133)	110 (82.7)	23 (17.3)	0.82 (0.37)	126 (94.7)	7 (5.3)	0.94 (0.22)
Overall (N = 405)	323 (79.8)	82 (20.2)	0.79 (0.40)	382 (94.3)	23 (5.7)	0.94 (0.23)

## Data Availability

The data that support the findings of this study will be made available by the authors upon reasonable request.

## References

[1] Armstrong M., Morris C., Abraham C., Tarrant M. (2017). Interventions utilising contact with people with disabilities to improve children’s attitudes towards disability: A systematic review and meta-analysis. Disabil. Health J..

[2] Maftei A., Gherguț A., Roca D., Dănilă O. (2022). Transitioning from decades of segregation: Religiosity and the attitudes towards intellectual disability in Romania. J. Beliefs Values.

[3] Pașcalău-Vrabete A., Crăciun C., Băban A. (2021). Restricted mobility and unheard voices: Perceptions of accessibility and inclusion expressed on Romanian disability-specific blogs and forums. Disabil. Rehabil..

[4] Gherguț A. (2011). Education of Children with Special Needs in Romania: Attitudes and Experiences. Procedia Soc. Behav. Sci..

[5] Phillips S.D. (2012). Implications of EU accession for disability rights legislation and housing in Bulgaria, Romania, Croatia and the former Yugoslav Republic of Macedonia. J. Disabil. Policy Stud..

[6] Babik I., Gardner E.S. (2021). Factors affecting the perception of disability: A developmental perspective. Front. Psychol..

[7] Nowicki E.A., Sandieson R. (2002). A meta-analysis of school-age children’s attitudes towards persons with physical or intellectual disabilities. Int. J. Disabil. Dev. Educ..

[8] Kayama M. (2017). Development of children’s understandings of physical disabilities and stigmatization in a Japanese cultural context: Reflections of children in second through sixth grades. Child. Youth Serv. Rev..

[9] Freer J.R. (2021). Students’ attitudes toward disability: A systematic literature review (2012–2019). Int. J. Incl. Educ..

[10] Armstrong M., Morris C., Abraham C., Ukoumunne O.C., Tarrant M. (2016). Children’s contact with people with disabilities and their attitudes towards disability: A cross-sectional study. Disabil. Rehabil..

[11] Maftei A., Ghergut A. (2021). Are Attitudes Towards Disability Different When We Refer to Children Versus Adults?. Int. J. Disabil. Dev. Educ..

[12] Alnahdi G.H. (2019). The positive impact of including students with intellectual disabilities in schools: Children’s attitudes towards peers with disabilities in Saudi Arabia. Res. Dev. Disabil..

[13] Magnusson D.M., Cal F., Boissonnault J.S. (2017). Influence of a short-term disability awareness program on knowledge and attitudes of school-aged children in Southern Belize: Results of a community-university partnership. Phys. Ther..

[14] Magiati I., Dockrell J.E., Logotheti A.E., Magiati I., Dockrell J.E., Logotheti A.E. (2002). Young children’s understanding of disabilities: The influence of development, context, and cognition. J. Appl. Dev. Psychol..

[15] Kang S.K., Inzlicht M., Kang S.K., Inzlicht M. (2012). Stigma building blocks: How instruction and experience teach children about rejection by outgroups. Personal. Social Psychol. Bull..

[16] Brinkman B.G., Jedinak A., Rosen L.A., Zimmerman T.S., Brinkman B.G., Jedinak A., Rosen L.A., Zimmerman T.S. (2011). Teaching children fairness: Decreasing gender prejudice among children. Anal. Soc. Issues Public Policy.

[17] FitzRoy S., Rutland A. (2010). Learning to control ethnic intergroup bias in childhood. Eur. J. Soc. Psychol..

[18] Skinner A.L., Meltzoff A.N. (2019). Childhood experiences and intergroup biases among children. Soc. Issues Policy Rev..

[19] Wang Z., Xu X., Han Q., Chen Y., Jiang J., Xin-Ni G. (2021). Factors associated with public attitudes towards persons with disabilities: A systematic review. BMC Public Health.

[20] Sahin H., Akyol A.D. (2010). Evaluation of nursing and medical students’ attitudes towards people with disabilities. J. Clin. Nurs..

[21] Hong S.Y., Kwon K.A., Jeon H.J. (2014). Children’s attitudes towards peers with disabilities: Associations with personal and parental factors. Infant Child Dev..

[22] Pelleboer-Gunnink H.A., Van Oorsouw W., Van Weeghel J., Embregts P. (2017). Mainstream health professionals’ stigmatising attitudes towards people with intellectual disabilities: A systematic review. J. Intellect. Disabil. Res..

[23] (2020). Paseka A; Schwab, S. Parents’ attitudes towards inclusive education and their perceptions of inclusive teaching practices and resources. Eur. J. Spec. Needs Educ..

[24] Jury M., Perrin A.L., Rohmer O., Desombre C. (2021). Attitudes toward inclusive education: An exploration of the interaction between teachers’ status and students’ type of disability within the French context. Front. Educ..

[25] Royal G.P., Roberts C.M. (1987). Students’ perceptions of and attitudes toward disabilities: A comparison of twenty conditions. J. Clin. Child Psychol..

[26] Nabors L., Keyes L. (1995). Preschoolers’ reasons for accepting peers with and without disabilities. J. Dev. Phys. Disabil..

[27] De Laat S., Freriksen E., Vervloed M.P. (2013). Attitudes of children and adolescents toward persons who are deaf, blind, paralyzed or intellectually disabled. Res. Dev. Disabil..

[28] Carvalho M., Perry A., Bebko J., Minnes P. (2014). Brief report: Social inclusion of Ontario children with developmental disabilities in community settings. J. Dev. Disabil..

[29] Eisenberg N., Fabes R.A., Spinrad T.L., Damon W., Lerner R.M., Eisenberg N. (2006). Prosocial Orientation. Handbook of Child Psychology.

[30] Portt E., Person S., Person B., Rawana E., Brownlee K. (2020). Empathy and positive aspects of adolescent peer relationships: A scoping review. J. Child Family Stud..

[31] Warrier V., Grasby K.L., Uzefovsky F., Toro R., Smith P., Chakrabarti B., Khadake J., Mawbey-Adamson E., Litterman N., Hottenga J.J. (2018). Genome-wide meta-analysis of cognitive empathy: Heritability, and correlates with sex, neuropsychiatric conditions and cognition. Mol. Psychiatry.

[32] Singer T. (2006). The neuronal basis and ontogeny of empathy and mind reading: Review of literature and implications for future research. Neurosci. Biobehav. Rev..

[33] Mestre M.V., Carlo G., Samper P., Malonda E., Mestre A.L. (2019). Bidirectional relations among empathy-related traits, prosocial moral reasoning, and prosocial behaviors. Soc. Dev..

[34] Stocks E.L., Lishner D.A., Decker S.K. (2009). Altruism or psychological escape: Why does empathy promote prosocial behavior?. Eur. J. Soc. Psychol..

[35] Yu S., Ostrosky M.M., Fowler S.A. (2015). The Relationship Between Preschoolers’ Attitudes and Play Behaviors Toward Classmates with Disabilities. Top. Early Child. Spec. Educ..

[36] Abdel-Khalek A.M., Halloway F. (2016). Introduction to the Psychology of Self-Esteem. Self-Esteem: Perspectives, Influences, and Improvement Strategies.

[37] Denissen J.J., Penke L., Schmitt D.P., van Aken M.A. (2008). Self-esteem reactions to social interactions: Evidence for sociometer mechanisms across days, people, and nations. J Pers Soc Psychol..

[38] Green L.M., Missotten L., Tone E.B., Luyckx K. (2018). Empathy, depressive symptoms, and self-esteem in adolescence: The moderating role of the mother–adolescent relationship. J. Child Family Stud..

[39] Fiske S.T., Cuddy A.J., Glick P., Xu J. (2018). A Model of (Often Mixed) Stereotype Content: Competence and Warmth Respectively Follow from Perceived Status and Competition. Social Cognition.

[40] Rohmer O., Louvet E. (2018). Implicit stereotyping against people with disability. Group Process. Intergroup Relat..

[41] Oldmeadow J.A., Fiske S.T. (2010). Social status and the pursuit of positive social identity: Systematic domains of intergroup differentiation and discrimination for high-and low-status groups. Group Process. Intergroup Relat..

[42] Smith L.A., Williams J.M. (2004). Children’s understanding of the causal origins of disability. J. Cogn. Dev..

[43] Rosenbaum P.L., Armstrong R.W., King S.M. (1988). Determinants of children’s attitudes toward disability: A review of evidence. Childrens Health Care.

[44] Vignes C., Coley N., Grandjean H., Godeau E., Arnaud C. (2008). Measuring children’s attitudes towards peers with disabilities: A review of instruments. Dev. Med. Child Neurol..

[45] Godeau E., Vignes C., Sentenac M., Ehlinger V., Navarro F., Grandjean H., Arnaud C. (2010). Improving attitudes towards children with disabilities in a school context: A cluster randomized intervention study. Dev. Med. Child Neurol..

[46] Bossaert G., Petry K. (2013). Factorial validity of the Chedoke-McMaster attitudes towards children with handicaps scale (CATCH). Res. Dev. Disabil..

[47] De Boer A., Pijl S.J., Post W., Minnaert A. (2012). Which variables relate to the attitudes of teachers, parents and peers towards students with special educational needs in regular education?. Educ. Stud..

[48] Vossen H.G.M., Piotrowski J.T., Valkenburg P.M. (2015). Development of the Adolescent Measure of Empathy and Sympathy (AMES). Personal. Individ. Differ..

[49] Rosenberg M. (1965). Society and the Adolescent Self-Image.

[50] Tyupa S. (2011). A Theoretical Framework for Back-Translation as a Quality Assessment Tool. New Voices Transl. Stud..

[51] Hayes A.F. (2013). Mediation, Moderation, and Conditional Process Analysis. Introduction to Mediation, Moderation, and Conditional Process Analysis: A Regression-Based Approach Edition.

[52] George D., Mallery P. (2010). SPSS for Windows Step by Step. A Simple Study Guide and Reference (10th Update) GEN.

